# The evaluation of three treatments for plantar callus: a three-armed randomised, comparative trial using biophysical outcome measures

**DOI:** 10.1186/s13063-016-1377-2

**Published:** 2016-05-17

**Authors:** Farina Hashmi, Christopher J. Nester, Ciaran R.F. Wright, Sharon Lam

**Affiliations:** School of Health Sciences, Brian Blatchford Building, University of Salford, Manchester, M6 6PU UK; Reckitt Benckiser, Hull, UK

**Keywords:** Hydration, Texture, Elasticity, Potassium hydroxide, Trichloroacetic acid, Podiatry, Foot skin

## Abstract

**Background:**

Callus is one of the most common foot skin complaints experienced by people of all ages. These painful and unsightly lesions often result in disability. The ‘gold standard’ of treatment is scalpel debridement by a trained specialist; however, people also seek over-the-counter remedies. There is a lack of clinical evidence for the efficacy of such products, which makes selection by patients and practitioners difficult.

**Methods:**

This randomised, three-armed, parallel, comparative trial aimed to test the efficacy of two home treatments for plantar callus using novel, objective outcome measures (skin hydration using the capacitance method; elasticity using negative pressure application; and surface texture using imaging). Additional outcome measures were: size of callus, quality of life (Foot Health Status Questionnaire) and self-reported participant satisfaction and compliance. The results were compared to a podiatry treatment. Participants were randomly allocated to one of three groups: potassium hydroxide (KOH, 40 %); trichloroacetic acid (TCA); and podiatry treatment. Participants were followed for 3 weeks after their initial intervention appointment (days 7, 14 and 21). The primary outcomes were the change from baseline in callus hydration, elasticity, texture, and size at each of the three time points. The secondary outcomes where: change in quality of life 21 days after treatment; resolution of calluses via visual inspection; and participant compliance and perception.

**Results:**

Forty-six participants (61 ft) with plantar calluses were recruited. The podiatry treatment showed immediate and significant changes in all objective outcomes, associated foot pain and function (*p* <0.01). Lesser changes in skin quality and perceived pain and functional benefits occurred with TCA and KOH over 21 days.

**Conclusions:**

This is the first study where objective outcome measures have been used to measure changes in the nature of skin in response to callus treatments. We found significant differences in plantar callus in response to podiatry and two home treatments. The podiatry treatment showed immediate and significant changes in skin and associated foot pain and function. Lesser, but sometimes comparable, changes in skin and perceived pain and functional benefits occurred with TCA and KOH over 21 days.

**Trial registration:**

ISRCTN14751843: date of registration: 30 April 2015.

**Electronic supplementary material:**

The online version of this article (doi:10.1186/s13063-016-1377-2) contains supplementary material, which is available to authorized users.

## Background

Foot callus is a hyperkeratotic skin lesion that commonly develops on the plantar surface of the forefoot in response to compression, friction and shearing forces [1–3]. Being the most common foot skin complaint in people of all ages, many seek advice and treatment from podiatrists [4]. Calluses are considered to be a common cause of foot pain [5, 6] and the secondary complications of these lesions can have a detrimental impact on the functional status of the foot in the elderly [7–11] as well as adding to the risk of ulceration in people with diabetes [12].

Currently, the ‘gold standard’ of treatment for calluses is scalpel debridement [1, 2, 9] with the regular application of topical keratolytics. Physical removal of callus has been shown to improve quality of life (QoL) and pain outcomes [13] although it is not clear whether this is the case for older people [14]. Despite the initial reasons for seeking treatment, the need for effective off-the-shelf topical treatments are warranted, as regularly clinical treatments can prove costly.

Caustic and acidic compounds such as potassium hydroxide (KOH) and trichloroacetic acid (TCA) are common keratolytics used in relatively low concentrations that are known to digest epidermal keratin [15, 16]. More recently these compounds have been incorporated in greater concentrations in callus removal preparations. The little evidence that is available as to the efficacy of these treatments is based on the subjective opinions of the users rather than on objective quantitative measures of skin properties. The measurement of the improvement of the structure and function of foot skin and callus would strengthen confidence in the selection of appropriate and effective treatments. Developments in measurement technology to objectively quantify changes in skin hydration, elasticity, texture have recently been applied successfully to the plantar surface of the foot [17, 18]. These papers report the high level of reliability of these measures for evaluating plantar skin and their ability to identify differences between normal and hyperkeratotic plantar skin, such as heel fissures, calluses and corns. This now presents an opportunity to objectively quantify for the first time the effects of various treatments of plantar callus.

The aim of this study was to evaluate the effectiveness of two common keratolytic compounds used for the removal of plantar callus: potassium hydroxide (KOH) and trichloroacetic acid (TCA). The measures taken were: skin surface hydration using capacitance; full skin elasticity using the application of negative pressure; and skin surface texture using high-resolution imaging. The outcomes were compared to the ‘gold standard’ of a typical clinical podiatry treatment. Hypothesis (equivalence framework): there is a significant difference, in objective outcome measures, between KOH/TCA therapies and podiatry treatment.

## Methods

### Study design

This study was a randomised, three-armed, parallel, comparative trial. Recruitment occurred from August 2012 to October 2013. Participants were randomly allocated to receive one of three treatments: (1) KOH-based treatment, (2) TCA-based treatment, and (3) clinical podiatry treatment. The study protocol was reviewed and approved by the University of Salford, Department of Health Sciences Ethical Approval Committee (application number HSCR12/55) and conforms to the provisions in the Declaration of Helsinki. The protocol was registered in the ISRCTN registry (ID: ISRCTN14751843). All participants provided written informed consent prior to taking part. The trial was conducted at one academic clinical site (Podiatry Clinical Facility, University of Salford, Manchester, UK).

### Participant recruitment

Participants were recruited via flyers within the university and advertisements in the local newspaper. Volunteers were included if they were aged 18 years and older and had plantar callus that had not been treated in the previous 6 weeks. This was assessed by a registered podiatrist. Both men and women were included.

Participants were excluded if they had any foot skin disorders such as infections (e.g. athlete’s foot), dermatitis, psoriasis, unhealed skin wounds, ulcers or blisters. Any participant with a known systematic disease, including peripheral vascular disease or a musculoskeletal disorder of the foot or ankle, rheumatoid arthritis or diabetes, was excluded. Participants were asked if they were allergic to any topical preparations. Participants were also deemed ineligible if they were unable to reach their feet. Volunteers were asked not to use any foot products (e.g. creams and powders) during the study and for 48 h prior to the screening appointment. If they were unable to stop using a product due to medical or personal reasons, they were not included in the study.

### Randomisation and blinding

The random allocation sequence was generated in one block of 90 (30 for each treatment group) with the knowledge that fewer participants than this would be recruited. The allocations were concealed from the investigator enrolling participants by folding the opaque paper with the allocations on and placing them in sealed envelopes (as per SPIRIT 2013 Checklist: Recommended items to address in a clinical trial protocol and related documents [19] and Consolidated Standards of Reporting Trials (CONSORT) Statement [20], see Additional files 1 and 2). Participants with multiple calluses were asked to nominate one callus pre randomisation, usually the most uncomfortable or largest, which was classed as the ‘index’ callus.

Due to the physical nature of the treatments, and their effects on the skin, the participants and the investigator could not be blinded to them. With regard to the outcome measures, these were self-reported outcome measures and objective skin evaluation measures apart from the evaluation of the photographs which have the potential to be subject to assessor bias. Therefore, we mitigated for this by ensuring that the images were coded before quantitative evaluation of the size of the calluses was carried out and, more importantly, there was a sufficient time period between the coding, analysis and uncoding processes. This was typically 14 days, and limited the ability of the assessor to associate a photograph with a specific patient and treatment group. In addition, the photographs were of the forefoot only and, therefore, did not have any features that allowed the participant to be identified.

### Interventions

The KOH (40 %, Balsan® Callus Removal Lotion) and TCA (concentration unknown, Wartner® Corn and Callus Removal Pen) treatments are available as over-the-counter remedies. Participants were advised to follow the manufacturer’s instructions. The KOH product was applied by the participants at weekly intervals in the research clinic. The corrosive action of KOH on relatively healthy skin is a known risk factor. According to the manufacturer’s instructions regarding the application of the product, there was a risk that the KOH could come in contact with healthy skin, therefore increasing the risk of corrosive damage. To comply with ethical requirements, application by the participant in a clinical environment was deemed more appropriate. The treatment involved soaking a cotton wool pad with the liquid and placing it in contact with the callused skin for 15 min, followed by manually scraping the softened tissue with a blunt spatula. The participants were given an instruction leaflet provided by the product manufacturer after randomisation into the treatment group. In the case of the TCA treatment, participants applied the liquid to the callused skin after soaking the foot in water for 10 min and then lightly rubbing the surface of the callus with an emery board. The participants were advised to do this for four consecutive days followed by 4 days of not using the treatment and then 4 days using the treatment, etc.

The participants in the podiatry group received a typical clinical podiatry treatment on the first day of the study (day D0), after which no treatment was given at follow-up appointments. The treatment involved sharp debridement (using a scalpel) followed by the use of a fine sandpaper sanding disc (Moore’s disc) on an electrically powered rotating device (20,000 rpm). The use of the sanding disc was necessary to provide a seamless transition and, therefore, a clear view of the area of skin at the edge of the callus, i.e. between the removed callus area and the adjacent normal skin. This was necessary in some (not all) cases depending upon the geometry of the callus surface post debridement, which varies naturally due to skin type. This clearer view ensured correct placement of measurement probes on the uncallused skin site. Nonetheless, the amount of callus removed using this method was very small (less than 1 mm). Also, the measures were taken from the centre of the callus and, therefore, the impact of filing with the disc would be minimal on the outcome measures.

### Outcome measures

The primary outcome decided a priori was the change in hydration, elasticity, skin surface texture and callus size at all time points (D7, D14 and D21) compared to D0 for the KOH and TCA groups and compared to D0 post treatment for the podiatry group. The secondary outcome measures were: (1) change in QoL before and 21 days after treatment, (2) change in biophysical outcome measures on D7, D14 and D21 in the podiatry treatment group.

#### Subjective outcome measures

The number of calluses resolved at D21 for the KOH and the TCA groups and D0 immediately after treatment for the podiatry group were noted. Resolution was defined as the absence of index callus on visual inspection of the skin.

A validated QoL questionnaire (the *Foot Health Status Questionnaire*, *FHSQ*) was completed by each participant [21] on D0 and D21 of the trial. The FHSQ has been widely used in foot pain research and provides a score for overall foot health-related QoL. This paper reports the separate scores for *foot pain*, *foot function*, and *general foot health* domains. For appropriate interpretation of the results the minimal important difference (MID) was considered, where the MID for *pain* was 13 points and 7 points for *foot function.* The MID for the *general foot health* domain was 0 points [22].

#### Biophysical outcome measures and protocols

Prior to performing measurements, participants’ foot skin was allowed to acclimatise to room temperature and humidity conditions for at least 15 min. The participant sat on a plinth with legs extended and the plantar aspects of both feet facing the investigator.

##### Marking of the callus and control skin sites

Using a ruler the callus plaque was bisected along the horizontal and vertical axes and the centre was marked. An unaffected skin site in the region of the plantar aspect of the fifth metatarsal base was selected to be the control site. A detailed description of the reliability of the marking of the skin is described in Hashmi et al. [18].

##### Digital image capture

Photographs of the calluses were taken at all data collection appointments using a digital camera (Canon PowerShot SX210 IS, 14.1 mega pixels). A standard protocol was used for every photograph captured. A white card was placed behind the foot before capturing the image. Each foot was positioned so that the callus was central to the camera’s field of view. The camera was placed at a distance of approximately 10 cm away from the callus. All attempts were made to ensure that the front of the camera was perpendicular to the foot. The same lighting was used for every data collection session. The photographs were used for the subjective assessment of change in callus size and also for measuring callus size.

##### Skin measurement devices

The devices used were the: Corneometer® CM 825, Cutometer® 580 MPA and Visioscan® VC 98 (Courage-Khazaka, Cologne, Germany). These measured hydration, elasticity and skin surface texture respectively. Intra and intertester reliability and the standard error of measurement (SEM) for these devices on foot skin have been confirmed in previous work [18].

##### Callus area calculations

The approximate area of each callus was calculated using the following equations: π*r*^2^ for circular plaques and π*ab* for oval plaques (where *r* = radius, *a* = first radius of oval and *b* = second radius of oval).

#### Participant perception and compliance

##### Participant perception

Participants were asked, on D21 for the KOH and TCA groups and immediately after podiatry treatment of D0, whether they thought that their callus had improved in appearance according to the following Likert scale: a great deal, much, somewhat, a little, and not at all.

##### Intervention use

Treatment compliance was monitored via questionnaires provided at each measurement appointment regarding the number of times the treatment was not used over the 7 days preceding the measurement interval.

#### Collection and management of data

Baseline data were collected at the clinical appointment prior to randomisation. All the outcome measures subsequent to baseline were completed on the return appointments on D7, D14 and D21. At regular intervals during the study, a representative of the sponsor’s team visited the study centre. At each monitoring visit, the chief investigator and the monitor reviewed the study progress, compliance to the study protocol, and any emergent problems (e.g. adverse events or recruitment issues). The academic research team (Trial Management Group) held regular meetings, independent from the sponsor, throughout the study period to monitor: unblinded baseline data, safety of the participants, and any ethical issues.

All data were treated with the strictest confidence and in accordance with the Data Protection Act 1998. Paper documents were retained in a secure cabinet in a locked room both during and after trial completion. During the course of the trial any personal identifiable paper records were stored separately from anonymised paper records. Once the study was complete, data were archived on the University of Salford campus, with access restricted to the research team only. All electronic records will be stored indefinitely on a secure, password-protected server within the University of Salford.

### Sample size and statistical analysis

A sample size of 60 (i.e. 20 callus lesions per group) provided an 80 % probability of detecting a difference between interventions of 5 arbitrary units (AU) (standard deviation 3 AU) of skin surface hydration readings using the Corneometer® by D21 (*ɑ* = 0.05). These estimates were taken from one published study that focussed on measuring the efficacy of cream treatment for dry heel skin over a period of 14 days [13]. There are no published studies that have tested the effects of callus treatments using the outcome measures used in our study.

The results were analysed and reported in two ways: (1) all data were analysed by intention-to-treat analysis. For all missing data a multiple imputation method using Amellia II software was used to generate missing at random data [23]. Continuous data were tested initially for deviation from the assumption of a normal distribution using the Shapiro-Wilk test of normality. The majority of the variables were not normally distributed; therefore, non-parametric inferential statistical tests were used; and (2) the number of resolved calluses according to visual inspection of the photographs was expressed as an odds ratio (OR). Statistical significance was set at *p* ≤0.01.

In the situation where individual questions within the FHSQ remained unanswered, the middle value on the response scale was assumed. This was done provided no more than 50 % of the responses for that particular subdomain were missing. Where more than 50 % of the responses in a subdomain were missing the total score for that subdomain was not calculated.

### Progression of participant through the trial

**Fig. 1:**
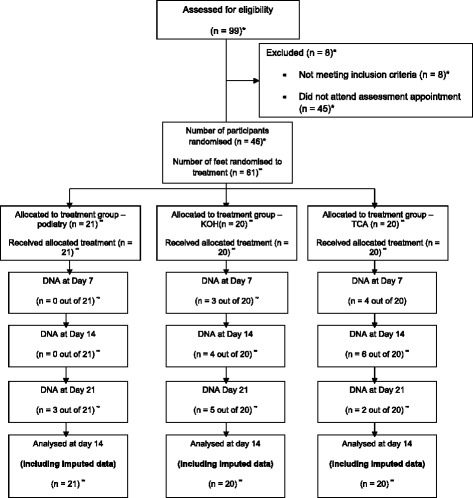
Progression of the participants through the trial. Primary end point at D21. Study design using CONSORT Statement guidelines [20]. *n represents the number of people, ^∞^ *n* represents the number of feet, *DNA* did not attend

Forty-six people were recruited to the study, of which 15 had callus on both feet. Thirty-one people had callus on one foot only. Therefore, a total of 61 callus lesions and feet were evaluated. The progression of the participants through the trial is presented in Fig. 1. There was a relatively small amount of missing data that required imputation. Ten participants were lost to follow-up by D21. Non-attendance figures for D7 and D14 are stated in Fig. 1.

### Adverse event reporting

Adverse events (including assessment of the seriousness, causality, expectedness, and intensity of the event/reaction) were recorded on an adverse event form, as appropriate. Participants were asked at each trial visit whether they had had any problems following their treatment and if any adverse events had occurred. The adverse event reaction reporting period for this trial began when the participant was randomised into the study and ended 21 days after the date of randomisation.

Potential complications associated with the treatments were pain or skin irritation. The criteria for discontinuing the allocated interventions for a given trial participant were: participant request, worsening of symptoms (such as pain), or irritant reactions to the topical therapies.

## Results

There were no adverse events. The majority of the reasons for non-attendance on D7 and D14 were related to participant personal matters and were not related to the interventions. The reasons for the 10 people not attending the D21 appointments are not known.

### Baseline data

The average room temperature and relative humidity were 23.6 ± 1.0 °C and 53.1 ± 8.4 % respectively. Table 1 provides the baseline characteristics of participants including the FHSQ data before randomisation. Sixty-one feet were randomised into the three treatment groups. Age ranged from 23 to 78 years (median 48, interquartile range (IQR) 24 years) and all groups were primarily female (84 %, 51/61). The three groups were balanced with respect to demographic data and there was no significant difference between the groups. With regards to baseline FHSQ data there were also no statistically significant differences between the groups for all the subdomains. However, when considering MID between the groups the *pain* domain (MID = 13 points) for the podiatry group score was significantly lower than the KOH and TCA groups. The *foot function* (MID = 7 points) and *general foot health* (MID = 0 points) domain scores all differed significantly between groups at baseline. Baseline data for the physical properties of the skin are reported in Table 2. The data in the groups are not as well-balanced as those of the demographic data and, therefore, the subsequent results are described as differences from baseline as opposed to absolute values.

**Table 1:** Baseline characteristics of all participants

Variable	Type of intervention		
	Podiatry (*n* = 21)	KOH (*n* = 20)	TCA (*n* = 20)
Age (years)			
Median	52	54	43.5
Min, max	24, 68	23, 73	24, 68
IQR	24.5	26.75	19
Sex (% female)	86	85	80
Height (m)			
Median	1.57	1.65	1.47
Min, max	1.42, 1.98	1.47, 1.88	1.49, 1.88
IQR	0.23	0.11	0.18
Weight (kg)			
Median	78.25	68.50	84.5
Min, max	46.3, 111.6	46.3, 101.6	57.0, 95.3
IQR	36.07	21	18.25
BMI (kg/m^2^)			
Median	27.5	26	29
Min, max	19, 41	20, 36	22, 41
IQR	9.75	4.75	6.25
FHSQ score: range from 0–100			
Foot pain			
Median	54.06	80.00	81.25
Min, max	12.5, 100	35.63, 100	25, 100
IQR	60.47	31.72	50.31
Foot function			
Median	84.38	100	93.75
Min, max	25, 100	68.75, 100	43.75, 100
IQR	48.44	31.72	25.00
General foot health			
Median	33.75	51.25	60
Min, max	0, 60	25, 100	0, 100
IQR	56.88	39.38	56.88

*BMI* body mass index, *FHSQ* Foot Health Status Questionnaire, *IQR* interquartile range, *KOH* potassium hydroxide, *TCA* trichloroacetic acid

**Table 2:** Baseline data for the podiatry, potassium hydroxide (KOH) and trichloroacetic acid (TCA) groups

	Skin sites					
	Podiatry (*n* = 21)		KOH (*n* = 20)		TCA (*n* = 20)	
	Callus	Control	Callus	Control	Callus	Control
Skin property						
Skin surface hydration (AU)						
Median	2.83	8.67	1.96	9.05	3.45	10.35
Min, max	0.00, 15.55	3.03, 22.0	0.20, 16.07	3.5, 50.41	0.48,13.24	0.36,28.37
IQR	3.46	7.98	1.96	8.10	3.07	10.02
Elastic properties of the skin (mm)						
Median	0.33	0.87	0.52	0.89	0.47	1.02
Min, max	0.00, 1.29	0.48, 1.42	0.00, 1.13	0.64, 1.40	0.04, 1.27	0.69, 1.39
IQR	0.59	0.26	0.58	0.23	0.56	0.22
Skin surface texture or scaliness (AU)						
Median	1.02	0.84	0.91	0.88	1.19	0.84
Min, max	0.52, 1.65	0.40, 2.89	0.52, 2.84	0.38, 2.81	0.65, 2.19	0.38, 2.46
IQR	0.50	1.49	0.57	0.88	0.52	1.00
Surface area [cm^2^]						
Median	3.42		2.02		2.16	
Min, max	0.44, 9.42		0.28, 9.54		0.39, 5.50	
IQR	3.19		2.55		2.92	

*AU* arbitrary units, *IQR* interquartile range

#### Primary outcomes

##### Intention-to-treat analyses

**Fig. 2:**
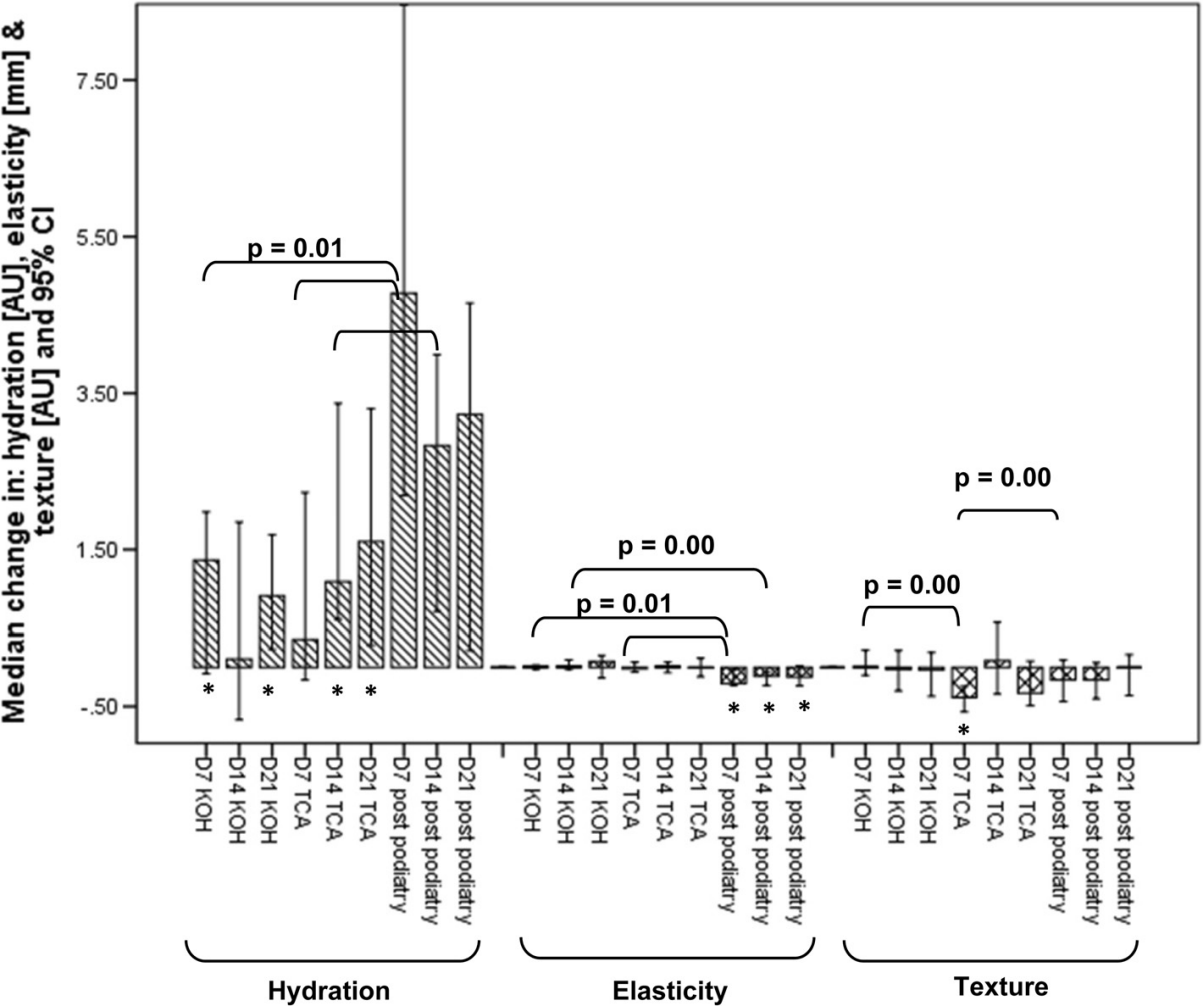
Median change in hydration, elasticity, and texture for plantar callus at D7, D14 and D21 for potassium hydroxide (KOH), trichloroacetic acid (TCA) and podiatry treatment groups. Change in a negative direction of hydration and elasticity data represents a decrease in hydration and elasticity respectively. Change in a negative direction of texture data represents an improvement in texture. *represents a significant difference (*p* ≤0.01) compared to baseline (the podiatry group baseline values were those taken post podiatry treatment on D0)

All four outcome measures showed statistically significant improvement compared to baseline measures immediately after podiatry treatment (*p* = 0.00). The calluses from both the KOH and TCA groups demonstrated an increase in hydration with time, apart from D14 where a reduction in hydration was noted in the KOH group. Increase in hydration compared to baseline was statistically significant on D7 and D21 in the KOH group and on D14 and D21 in the TCA group. Pairwise comparisons between the groups showed significant differences between the topical preparations and podiatry treatment. Improvement in skin elasticity (*p* = 0.04 and *p* = 0.91) and skin surface texture (*p* = 0.33 and *p* = 0.04) at D21 compared to baseline, was not significantly different for either group (Fig. 2). The callus plaques were significantly smaller in the KOH and TCA groups (*p* = 0.00). Pairwise comparisons (Table 3) showed no significant difference between the KOH and TCA groups in all outcome measures by D21. The podiatry treatment outcomes at D0 showed significant improvement in all outcomes compared to the KOH and TCA groups. There were no significant differences (*p* >0.01) in any measures at any time point at the control site (fifth metatarsal).

**Table 3:** Pairwise comparisons between the three groups for change in hydration, elasticity, skin surface texture and callus area by D21

	Median difference (*p* value)			
	Hydration (AU)	Elasticity (mm)	Skin surface texture (AU)	Callus area (cm^2)^
Podiatry vs KOH	4.31: 0.91 (0.00)	0.33: 0.08 (0.00)	0.00: −0.04 (0.00)	3.24: 0.81 (0.00)
Podiatry vs TCA	4.31: 1.60 (0.03)	0.33: −0.01 (0.00)	0.00: −0.25 (0.00)	3.24: 0.43 (0.00)
KOH vs TCA	0.91: 1.60 (0.28)	0.08: −0.01 (0.53)	−0.04: −0.25 (0.43)	0.81: 0.43 (0.97)

*AU* arbitrary units, *KOH* potassium hydroxide, *TCA* trichloroacetic acid

The KOH and TCA treatments demonstrated a gradual improvement in hydration and texture with time, apart from D14 where a reduction in hydration was noted in the KOH group and deterioration in texture in the TCA group. The elasticity of the skin improved gradually with time in the KOH group; however, the skin treated with TCA became less elastic with time. There was no significant difference between the groups at each time point with regards to hydration and elasticity measures; however, at D7 the skin texture had improved significantly in the TCA group compared to the KOH group (*p* = 0.00). The measurements taken from plantar skin after podiatry treatment showed a gradual increase in hydration and texture and a significant decrease in elasticity with time.

**Fig. 3:**
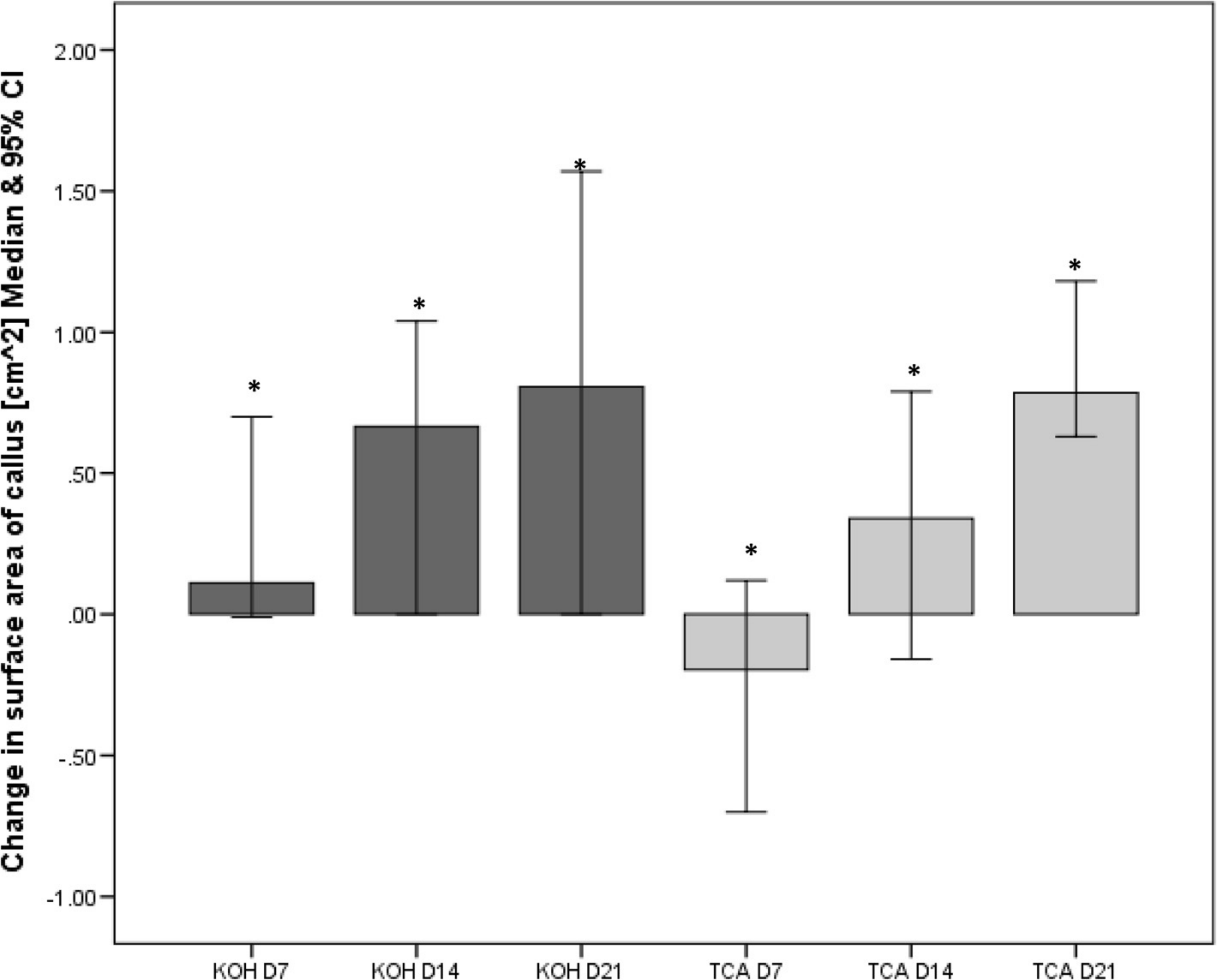
Change in surface area of the calluses in the potassium hydroxide (KOH) and trichloroacetic acid (TCA) treatment groups at D7, D14 and D21. Note that the greater the change in a positive direction the smaller the callus plaque and vice versa. *represents a significant difference compared to baseline (*p* = 0.00)

Figure 3 shows a significant reduction in callus surface area for D7, D14 and D21 for both treatment groups except for D7 in the TCA group. In this case the area of the callus increased (median increase in surface area: 0.20 mm^2^, IQR: 0.99 mm^2^). At D21 both groups showed a similar reduction in callus size: KOH and TCA median change in surface area: 0.81 cm^2^, 0.80 cm^2^ respectively.

### Subjective outcome measures

#### Callus resolution

##### Odds ratios

At 21 days post treatment 20 % (4/20) of the calluses had resolved in the KOH group compared to 0 % (0/20) in the TCA group (OR: 11.2, 95 % CI: 0.56 to 222.99, *p* = 0.11 in favour of the KOH group). The odds of a completely resolved callus were over 11 times greater for KOH treatment compared to the TCA at 21 days. However, these data did not reach significance. All calluses were resolved immediately after podiatry treatment on D0.

#### Quality of life measures

There were no statistically significant differences in QoL measures (foot pain, foot function and general foot health domains) at D21 post KOH and TCA treatments compared to baseline, nor immediately after podiatry treatment on D0 (Table 4). However, when considering the MID of the subdomains, all the treatment groups showed an improvement in *pain* of above 13 points (MID = 13); and an improvement of above 8 points (MID = 0) for *general foot health.* The change in *foot function* score was below the MID of 7 for all three groups.

**Table 4:** Change from baseline in the total scores from the Foot Health Status Questionnaire (FHSQ) at D21 (for KOH and TCA treatment groups and podiatry treatment on D0). A higher score is favourable

Variable	Podiatry (*n* = 21)	NaOH (*n* = 20)	TCA (*n* = 20)
Foot pain			
Change from baseline			
Median	18.13	13.44	13.34
Min, max	−26.88, 64.38	−6.25, 33.75	−2.11, 68.75
IQR	48.75	15.93	33.28
Pairwise comparisons	*P*		
Podiatry vs NaOH	0.80		
Podiatry vs TCA	0.83		
KOH vs TCA	0.45		
Foot function			
Change from baseline			
Median	6.25	0.00	5.73
Min, max	−12.50, 62.50	0.00, 31.25	−6.27, 56.25
IQR	21.88	12.50	23.44
Pairwise comparisons	*P*		
Podiatry vs NaOH	0.36		
Podiatry vs TCA	0.79		
KOH vs TCA	0.48		
General foot health			
Change from baseline			
Median	25.00	8.75	13.46
Min, max	−17.50, 92.50	−40.00, 100	−20.01, 72.50
IQR	63.13	45.63	41.30
Pairwise comparisons	*P*		
Podiatry vs NaOH	0.11		
Podiatry vs TCA	0.24		
KOH vs TCA	0.66		

*IQR* interquartile range, *KOH* potassium hydroxide, *NaOH* sodium hydroxide, *TCA* trichloroacetic acid

### Patient perception data

All participants (100 %, 21/21) who received the podiatry treatment perceived the treatment to have improved the condition of their skin ‘a great deal’ on D0. Eighty-two per cent (17/21) of the same group declared an improvement of either ‘much’ or ‘a great deal’ by D21. This compares to 65 % (13/20) in both KOH and TCA groups.

### Experience of use data

Only two people reported not using the TCA treatment according to the instructions on the pack between D14 and D21. One participant used the product twice rather than on four occasions, the other used it once.

## Discussion

This study aimed to measure and compare the structural and functional changes of plantar callused skin with use of two home treatments and in response to a clinical podiatry treatment. This is the first time that the efficacy of a range of callus treatments has been assessed using specific biophysical outcome measures taken at regular intervals over 21 days.

While all the outcome measures improved by D21 compared to baseline for all three treatment groups, only the podiatry group showed a statistically significant improvement in all skin biophysical measures, and only for immediately post treatment (*p* ≤0.01). Increased hydration and reduced callus surface area were statistically significantly different for all three treatment groups by D21 compared to baseline. However, despite the hydration increasing, elasticity did not change significantly for KOH and TCA groups. This would suggest that three things might be taking place: (1) the SC cells themselves have not shed from the bulk of the callus but have absorbed a degree of moisture as a result of the chemical actions of the compounds, and/or (2) a small amount of the very superficial layers of the SC have successfully desquamated from the bulk of the tissue but not enough to cause a significant change in the elasticity of the SC (the surface area data support this), or (3) exposure of some underlying callus tissue that is naturally more hydrated. The only way to appropriately test this hypothesis is to measure SC thickness as the treatments take effect. This is difficult to achieve using ultrasound as the densely packed SC of plantar skin acts as a barrier to the sound waves [24]. However, optical coherence tomography (OCT) produces high-resolution images of the stratum corneum akin to those of histology sections and could be a viable option for future research of this kind [25].

**Fig. 4:**
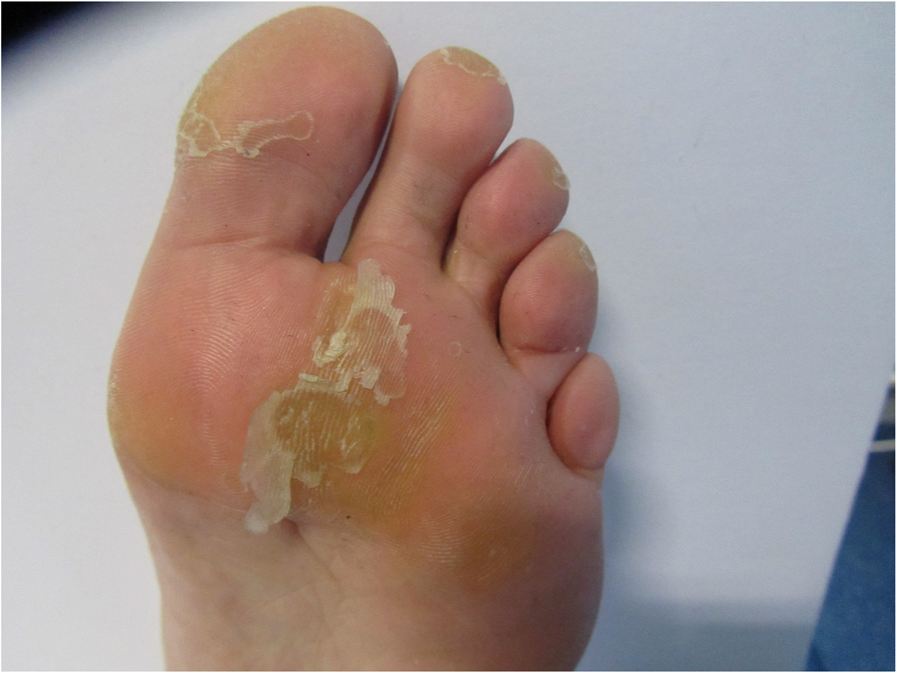
Photograph of a region of plantar callus after 7 days of TCA-based treatment use

In general the biophysical parameters for the KOH and TCA treatments showed similar improvement with time, except for skin surface texture. The KOH-treated callus showed consistent improvements throughout the trial whereas TCA-treated skin showed deterioration on D7 followed by improvements on D14 and D21. These data matched the progressive change in the visual appearance of the skin. After the first week of TCA the callus had loosely adhered sheets of SC (Fig. 4). This scaling skin may act as a barrier for TCA access to the underlying tissue and a more aggressive form of physical debridement is perhaps required.

The podiatry treatment group data have been interpreted in two ways. Firstly, by comparing the immediate improvement in the biophysical outcome measures to those at specific time points of the KOH and TCA groups. This was used to test how long it would take for the topical treatments to have an effect similar to that of podiatry treatment. With the exception of hydration in the TCA group on D14 and D21 the KOH and TCA data did not match that of the post-podiatry treatment measures. Secondly, comparing the quantitative measures of the skin after 21 days of topical treatment and 21 days after podiatry treatment showed no significant differences between the groups, and all groups reported clinically significant improvements in foot pain and general foot health. This suggests that in the absence of access to clinical treatment the use of home treatments may be of benefit to some sufferers.

The majority of the participants in all three groups reported that the appearance of the callus improved either by ‘much’ or ‘a great deal’ by the end of the trial period. Improvement in foot pain and general foot health perception had also improved in all the groups by greater than the recommended MID value, although the podiatry group scores were higher than those of the KOH and TCA group scores. The improvement in perception data thus matches that of the quantitative outcomes, implying that as the biophysical properties of the skin improve so do the perceptions of associated pain and general foot health.

All groups reported a reduction in pain greater than the MID of 13 points but since participants were not blinded to the treatments some placebo effect is possible. It is known that placebo effects due to expectation and conditioning are often acute responses (up to 2 weeks in pain studies) [5]. The 3-week trial time for our study may exceed these limits, but we cannot be sure. Landorf et al. [14] have previously shown no significant difference in terms of foot pain between podiatry and sham callus treatment groups.

The improvement in skin quality suggests that that the use of home treatments would be an appropriate adjunct to podiatry treatments in managing plantar callus: for example, between clinical appointments, and could perhaps help suffers lengthen the time between clinical appointments.

Prior clinical trials used observation and perception data to test the efficacy of callus treatments, relying on categorical data (i.e. the presence or absence of callus and evaluating changes in pain levels). The OR analysis of our data showed that the two home treatments were relatively ineffective whereas the objective measures indicate structural changes in specific qualities of the skin and that these were perceived by suffers.

The results of this study need to be viewed in light of a number of potential limitations. Firstly, this study was based at a single centre which poses the question of external validity. We are confident of the scientific rigour of the study but invite clinicians to compare the context of the trial with their own clinical situation. Secondly, due to the nature of the effects of the treatments on the skin participant blinding was not possible. This could have introduced bias with regard to patient-reported outcomes. We did, however, mitigate for assessor bias by ensuring appropriate blinding measures for the evaluation of callus size from the photographic data. Thirdly, the long-term benefits and the cost-effectiveness of the treatments have not been analysed in this study. Although this was initially considered, it was decided that the focus of the study should be primarily on testing for the immediate effects. The results would then inform a longer-term clinical and cost-effectiveness study which would consider callus recurrence rates. For example, podiatry treatment may provide exceptional short-term success but callus recurrence rate may imply that long-term success would likely be no better than the topical treatments. At this stage it may be assumed that podiatry treatment is more expensive compared to over-the-counter home treatments (given health care costs and models of care) but longer-term outcome may modify this view in terms of cost-effectiveness. Finally, as with all studies of this nature, the issue of monitoring patient compliance must be considered. The 21-day timescale was considered to be both practical given the burden on the users, appropriate with regard to consumer expectations of home treatments, and encouraging of compliance in terms of motivation and continued engagement. The TCA treatment was the only treatment used at home by the participants and TCA dispensers were not weighed at the end of the study, although this would only measure dispensed TCA amount rather than dose used. Compliance was evaluated via self-reported use data from the participants, which was further confirmed by the distinctive, flaky appearance of the callus during the review appointments. Figure 4 in the paper depicts the typical appearance of the callus during the TCA treatment period and, based on this, there were no grounds for suspicion of non-compliance.

## Conclusions

Using objective measures of skin structure and function, we found significant differences in plantar callus in response to podiatry and two home treatments. The podiatry treatment showed immediate and significant changes in skin and associated foot pain and function. Lesser but sometimes comparable changes in skin and perceived pain and functional benefits occurred with TCA and KOH over 21 days.

### Consent

Written informed consent was obtained from the patient(s) for publication of this manuscript and accompanying images. A copy of the written consent is available for review by the editor-in-chief of this journal.

## Additional files

Additional file 1: SPIRIT 2013 Checklist: Recommended items to address in a clinical trial protocol and related documents. The evaluation of three treatments for plantar callus: a three armed randomised, comparative trial using biophysical outcome measures. (DOC 121 kb) Additional file 2: CONSORT 2010 Checklist of information to include when reporting a randomised trial. The evaluation of three treatments for plantar callus: a three-armed randomised, comparative trial using biophysical outcome measures. (DOC 217 kb)

## Abbreviations

AU: arbitrary units
CI: confidence intervals
CONSORT: Consolidated Standards of Reporting Trials
D: day
FHSQ: Foot Health Status Questionnaire
IQR: interquartile range
ISRCTN: International Standard Randomised Controlled Trial Number
KOH: potassium hydroxide
MID: minimum important difference
OR: odds ratio
QoL: quality of life
SEM: standard error of measurement
TCA: trichloroacetic acid
OCT: optical coherence tomography
